# Teriflunomide and Epstein–Barr virus in a Spanish multiple sclerosis cohort: *in vivo* antiviral activity and clinical response

**DOI:** 10.3389/fimmu.2023.1248182

**Published:** 2023-09-29

**Authors:** María Inmaculada Domínguez-Mozo, Inés González-Suárez, Luisa María Villar, Lucienne Costa-Frossard, Noelia Villarrubia, Yolanda Aladro, Belén Pilo, Xavier Montalbán, Manuel Comabella, Ignacio Casanova-Peño, María Luisa Martínez-Ginés, Jose Manuel García-Domínguez, María Ángel García-Martínez, Rafael Arroyo, Roberto Álvarez-Lafuente

**Affiliations:** ^1^ Grupo de Investigación de Factores ambientales en enfermedades degenerativas, Instituto de Investigación Sanitaria del Hospital Clínico San Carlos (IdISSC), Red de Enfermedades Inflamatorias (REI), Madrid, Spain; ^2^ Unidad de Enfermedades Desmielinizantes, Hospital Álvaro Cunqueiro, Red de Enfermedades Inflamatorias (REI), Vigo, Spain; ^3^ Servicio de Inmunología, Hospital Universitario Ramón y Cajal, Red de Enfermedades Inflamatorias (REI), Madrid, Spain; ^4^ Servicio de Neurología, Hospital Universitario Ramón y Cajal, Red de Enfermedades Inflamatorias (REI), Madrid, Spain; ^5^ Servicio de Neurología, Hospital Universitario de Getafe, Getafe, Spain; ^6^ Servei de Neurologia-Neuroimmunologia, Centre d’Esclerosi Múltiple de Catalunya (Cemcat), Institut de Recerca Vall d’Hebron (VHIR), Hospital Universitari Vall d’Hebron, Universitat Autònoma de Barcelona, Barcelona, Spain; ^7^ Servicio de Neurología, Hospital Universitario de Torrejón, Torrejón de Ardoz, Spain; ^8^ Servicio de Neurología, Hospital General Universitario Gregorio Marañón/Red de Enfermedades Inflamatorias (REI), Madrid, Spain; ^9^ Departamento de Neurología, Hospital Universitario Quironsalud Madrid, Madrid, Spain

**Keywords:** multiple sclerosis, teriflunomide, biomarker, EDSS, EBV, HHV-6, NF-L, ELISA

## Abstract

**Background:**

Epstein–Barr virus (EBV) and human herpesvirus 6 (HHV-6) have been associated with multiple sclerosis (MS). Teriflunomide is an oral disease-modifying therapy approved for treatment of relapsing forms of MS. In the preclinical Theiler’s murine encephalitis virus model of MS, the drug demonstrated an increased rate of viral clearance versus the vehicle placebo. Furthermore, teriflunomide inhibits lytic EBV infection *in vitro*.

**Objective:**

1. To evaluate the humoral response against EBV and HHV-6 prior to teriflunomide treatment and 6 months later. 2. To correlate the variation in the humoral response against EBV and HHV-6 with the clinical and radiological response after 24 months of treatment with teriflunomide. 3. To analyze the utility of different demographic, clinical, radiological, and environmental data to identify early biomarkers of response to teriflunomide.

**Methods:**

A total of 101 MS patients (62 women; mean age: 43.4 years) with one serum prior to teriflunomide onset and another serum sample 6 months later were recruited. A total of 80 had been treated for at least 24 months, 13 had stopped teriflunomide before 24 months, and 8 were currently under teriflunomide therapy but with less than 24 months of follow-up. We analyzed the levels of the viral antibodies titers abovementioned in serum samples with ELISA commercial kits, and the levels of serum neurofilament light chain (Nf-L).

**Results:**

Antiviral antibody titers decreased for EBNA-1 IgG (74.3%), VCA IgG (69%), HHV-6 IgG (60.4%), and HHV-6 IgM (73.3%) after 6 months of teriflunomide. VCA IgG titers at baseline correlated with Nf-L levels measured at the same time (r = 0.221; p = 0.028) and 6 months later (r = 0.240; p = 0.017). We found that higher EBNA-1 titers (p = 0.001) and a higher age (p = 0.04) at baseline were associated with NEDA-3 conditions. Thus, 77.8% of patients with EBNA-1 >23.0 AU and >42.8 years (P50 values) were NEDA-3.

**Conclusion:**

Treatment with teriflunomide was associated with a reduction of the levels of IgG antibody titers against EBV and HHV-6. Furthermore, higher EBNA-1 IgG titers prior to teriflunomide initiation were associated with a better clinical response.

## 1 Introduction

Multiple sclerosis (MS) is a disease of unknown origin. Classically, it has been considered the result of the interaction of one or several environmental factors, which would act in the early stages of life on people predisposed from the genetic point of view. Many viruses have been associated with MS over the years. However, viruses of the *Herpesviridae* family, specifically Epstein–Barr virus (EBV), have accumulated the most evidence in recent years. The higher seroprevalence of EBV in patients with MS compared with healthy subjects has been widely described (1). In addition, the risk would increase in subjects who contract EBV in adulthood and develop infectious mononucleosis (IM) compared with those who are infected in childhood and do not develop IM (2). It has been hypothesized that dysregulation of EBV-infected B cells could induce leptomeningeal inflammation, which could contribute to subpial lesions and gray matter pathology in MS (3, 4). Thus, it has been observed that high levels of antibodies against EBV would be associated with an increase in the activity of lesions in MRI (5, 6), and greater development of brain atrophy, particularly of the cortical gray matter (6, 7). There are currently not many options to inhibit EBV replication, but previous *in vitro* studies have shown that teriflunomide could exhibit inhibitory activity on EBV (8).

Teriflunomide is an oral immunomodulator approved for the treatment of relapsing–remitting MS (RRMS) and clinically isolated syndrome (CIS). The efficacy and safety of teriflunomide in patients with RRMS were established in phase 2 (NCT01487096) and phase 3 (TEMSO-NCT00134563, TOWER-NCT00751881, and TENERE-NCT00883337) clinical trials (9–12) and in patients with CIS in the TOPIC study (NCT00622700) (13, 14). Recently, the findings from the phase 3 TERIS study (NCT03122652) showed that treatment significantly reduced the time to first clinical event in patients with radiologically isolated syndrome (RIS) (15). Teriflunomide reversibly inhibits the enzyme dihydroorotate dehydrogenase (DHODH), a key mitochondrial enzyme in the *de novo* synthesis of pyrimidines (16). It has been shown to have anti-inflammatory but also antiviral properties against different viruses: EBV (8), cytomegalovirus (17), herpes simplex virus 1 and 2 (18, 19), foot-and-mouth disease virus (20), and against poliovirus BK (21). It has been proposed that this antiviral effect would be achieved by inhibiting viral replication. Recent studies that describe antiviral compounds that act at the level of the *de novo* pyrimidine biosynthetic pathway, such as teriflunomide, raise the hypothesis that the mode of action of these molecules may be based on the inhibition of DHODH (22).

Thus, the objectives of this study were (1) to evaluate the *in vivo* antiviral activity of teriflunomide in MS patients through the evaluation of the variation of IgG antibody titers against EBNA-1 and VCA of EBV and IgG and IgM against HHV-6 between the baseline visit (before treatment initiation) and after 6 months of treatment with teriflunomide (2), to associate the variation of these titers with the clinical and radiological response after 24 months of treatment with teriflunomide, and (3) to search for early response biomarkers to teriflunomide.

## 2 Materials and methods

### 2.1 Design

A multicenter longitudinal study was performed. We recruited MS patients under the following inclusion criteria: a diagnostic of RRMS according to the McDonald criteria (23), with serum samples collected prior to the initiation of teriflunomide and 6 months later. Serum samples were collected in dry tubes; then, they were aliquoted and aliquots were frozen at −80°C.

### 2.2 Patients

They were recruited from the MS Units of the following hospitals: Hospital Clínico San Carlos, Hospital Universitario Ramón y Cajal, Hospital Universitario de Getafe, Hospital General Universitario Gregorio Marañón, Hospital Universitario de Torrejón, Hospital Álvaro Cunqueiro, and Hospital Universitario Vall d’Hebron, all of them in Spain.

### 2.3 Ethics statement

This study was approved by the local Ethic Committee of the Hospital Clínico San Carlos (Comité Ético de Investigación Clínica del Hospital Clínico San Carlos). All the patients recruited received and signed written informed consent. All experiments were performed in accordance with relevant guidelines and regulations.

### 2.4 Response criteria

Clinical response (absence of disability progression and relapses), NEDA-3 (no evidence of disease activity: patients without relapses, disability progression, and new T2 lesions or Gd+ lesions), and therapeutic failure (disability progression and/or ≥2 relapses) were evaluated after 2 years of follow-up. Relapses: a worsening of neurological impairment or an appearance of a new symptom or abnormality attributable to MS; they lasted at least 24 h and were preceded by a stability period of at least 1 month. Progression: according to EDSS score prior to teriflunomide initiation (1): EDSS = 0: ≥1.5 points (2); EDSS ≥1 and ≤5: ≥1 point (3); EDSS ≥5.5: ≥0.5 points. Magnetic resonance imaging (MRI) examination was performed within 1 month prior to teriflunomide initiation according to the protocols established in each center and 12 and 24 months later.

### 2.5 Viral antibody quantification

Serum samples were analyzed in an automated ELISA processing system (DS2, Dynex Technologies, USA) using the following commercial tests: anti-EBNA-1 and anti-VCA IgG (Trinity Biotech, USA) and anti-HHV-6A/B IgG and IgM (Vidia, Ltd., Czech Republic). Each sample was analyzed in duplicate for each test. Results were expressed in artificial units (AU), as we have previously described (24, 25).

### 2.6 sNfL quantification

Serum neurofilament light chain (sNfL) levels were quantified in a SR-X instrument (Quanterix, Lexington, MA) using the single-molecule array NF-light Advantage Kit technique (Quanterix, Billerica, MA) (26).

### 2.7 Statistical analysis

Differences in categorical variables were analyzed with the chi-square or two-tailed Fisher’s exact test. Differences in continuous variables were assessed with the Mann–Whitney U-test. A multiple logistic regression model was performed to eliminate the effect of the putative confounding variables in the univariate tests. p values below 0.05 were considered significant. Statistical analyses were performed with SPSS for Windows (Ver. 15.0) software (SPSS Inc.) and with GraphPad Prism 6.0 software (GraphPad Prism Inc., San Diego, CA).

## 3 Results

### 3.1 Patients recruited according to the inclusion criteria

**Figure 1:**
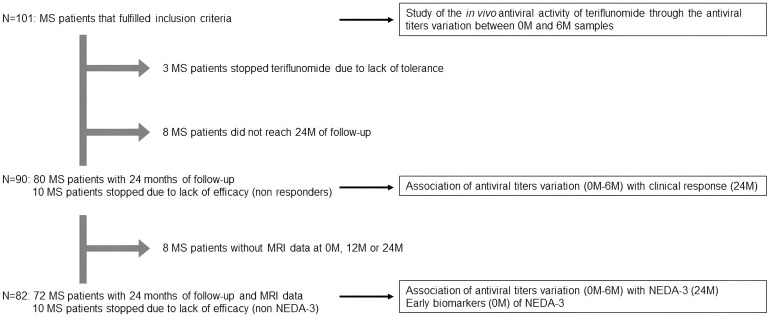
Study design. MS patients included in each one of the analysis.

As we can see in **Figure 1**, we recruited 101 MS patients. A total of 13 stopped teriflunomide before 24 months, 10 of which were due to lack of efficacy: four due to activity, three due to progression, and three due to activity and progression; all of them were considered non-clinical responders. The other three patients abandoned teriflunomide due to a lack of tolerance, and they were not included in the clinical response analysis. Other eight patients were under teriflunomide treatment, but they did not reach 24 months of follow-up yet. Thus, we performed the clinical response analysis with 90 patients. We also had radiological data (number of T2 lesions and gadolinium-enhanced lesions at 0, 12, and 24 months) from 82/90 patients to establish NEDA-3 conditions. Basal patient data are in **Table 1**.

**Table 1:** Basal characteristics of the patients recruited at the onset of teriflunomide treatment.

MS patients	101
Gender	
Males	26
Females	75
**Age (years, med (P25–P75))**	43.3 (38.3–49.0)
**Age at disease onset (years, med (P25–P75))**	30.6 (26.1–38.6)
**Disease duration at teriflunomide onset (months, med (P25–P75))**	101.0 (30.0–184.9)
**EDSS (med (P25–P75))**	1.5 (1.5–2.0)
**Relapses 2 years before (med (P25–P75))**	1.0 (0.0–1.0)
**T2 lesions at basal sample (% with less than 10 T2 lesions (n/N))**	14.9 (14/94)
**Gadolinium enhanced lesions at basal sample (med (P25-p75))**	0.0 (0.0-1.0)
MS patients without Gd+ lesions	65
MS patients with at least 1 Gd+ lesion	24

med: median; P25: 25th percentile; P75: 75th percentile; EDSS: Expanded Disability Status Scale.

### 3.2 Variation of antiviral IgG titers and Nf-L levels after 6 months with teriflunomide

**Figure 2:**
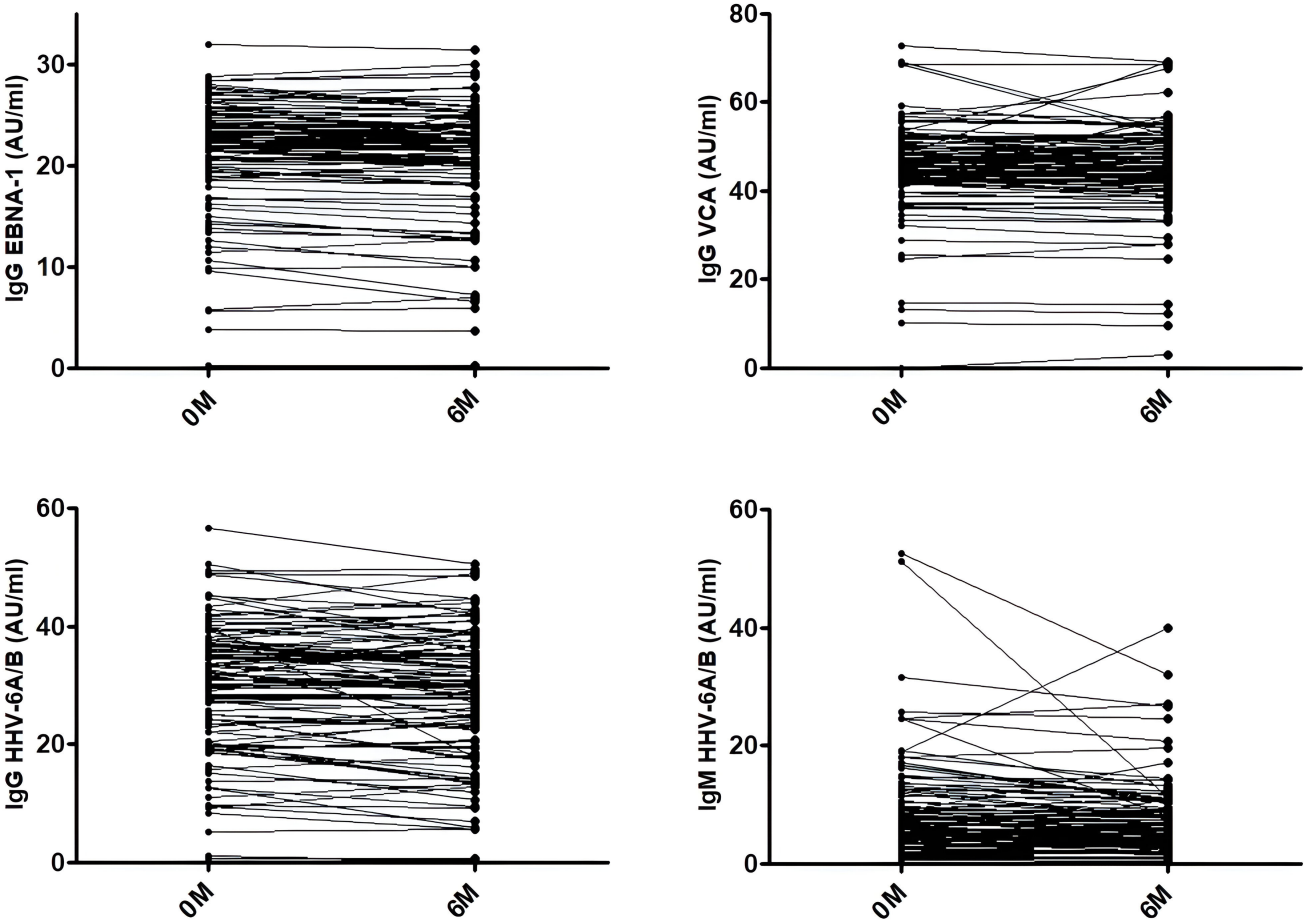
EBNA-1 IgG, VCA IgG, HHV-6 IgG, and HHV-6 IgM titers. Variation of the titers in each patient between samples collected prior to teriflunomide treatment (0M) and after 6 months of treatment (6M).

Antiviral antibody prevalences and titers in samples collected prior to teriflunomide initiation are in **Table 2**. After 6 months of teriflunomide treatment, antiviral antibody titers decreased in 61/101 (60.4%) MS patients for HHV-6 IgG, 75/101 (74.3%) for HHV-6 IgM, 74/101 (73.3%) for EBNA-1 IgG and 69/100 (69%) for VCA IgG (**Figure 2**). No statistically significant differences were found between both genders or between those that were above or below 43.4 years (median value) for any of the viruses.

**Table 2:** Serologies of the virus included in the study prior to the initiation of teriflunomide treatment.

	Total		Female		Male	
	n/N (%)	Mean titers (AU)	n/N (%)	Mean titers (AU)	n/N (%)	Mean titers (AU)
**HHV-6 IgG**	93/101 (92.1)	31.0	58/62 (93.5)	30.1	35/39 (89.7)	31.1
**HHV-6 IgM**	25/101 (24.8)	6.6	17/62 (27.4)	7.1	8/39 (20.5)	5.8
**EBNA-1 IgG**	94/101 (93.1)	22.2	58/62 (93.5)	21.7	36/39 (92.3)	23.0
**VCA IgG**	98/100* (98.0)	45.7	60/62 (96.8)	45.4	38/39 (97.4)	47.1

*There was a missing value.

**Figure 3:**
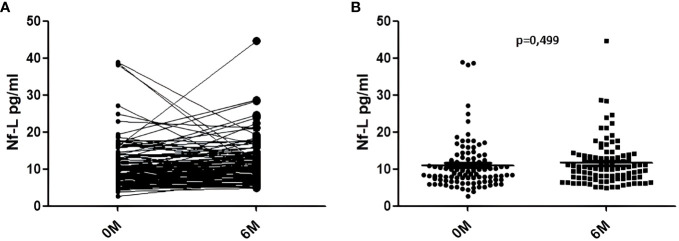
Nf-L levels in serum samples collected before teriflunomide initiation (0M) and after 6 months of treatment (6M). **(A)** Variation of the levels in each sample. **(B)** Comparison of the Nf-L levels at 0M and 6M.

We did not find any statistical significant difference between the levels of Nf-L prior to the initiation of teriflunomide treatment and after 6 months of therapy (11.0 ± 6.7 pg/ml vs. 11.6 ± 6.0; p = 0.499) (**Figure 3**).

**Figure 4:**
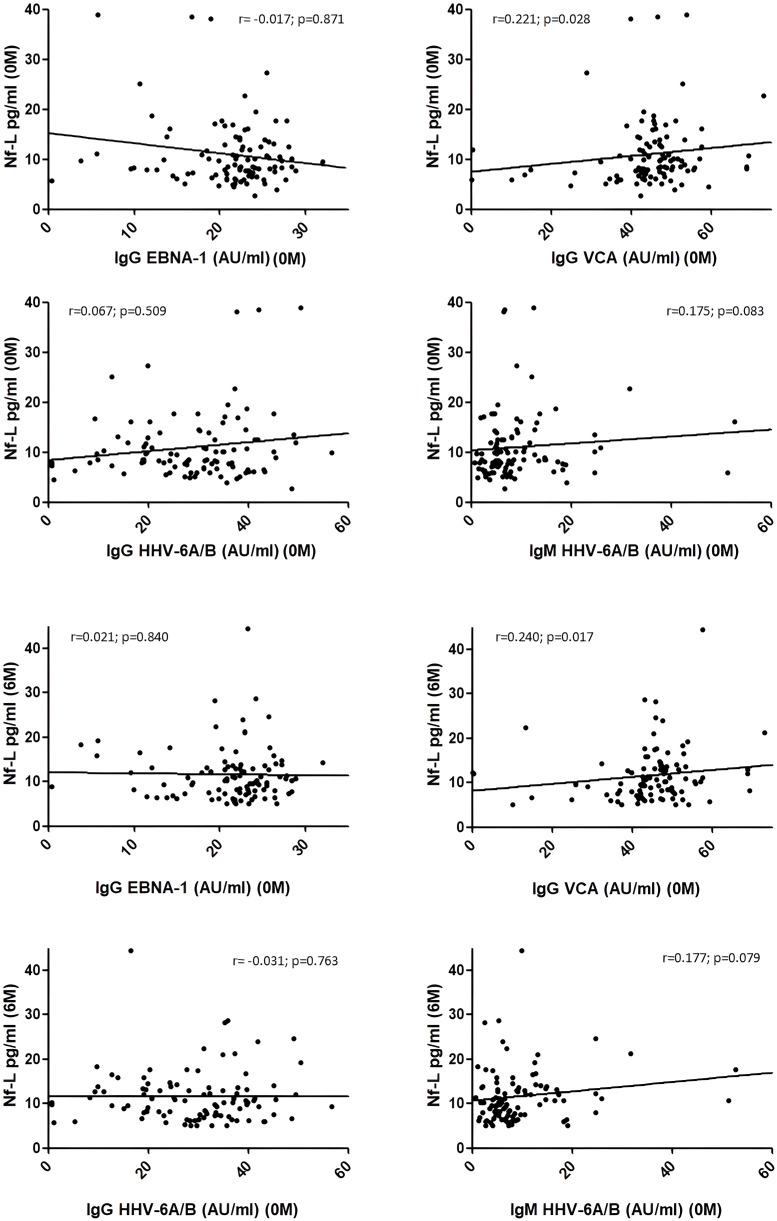
Correlation between Nf-L levels in serum samples collected before teriflunomide initiation (0M) and after 6 months of treatment (6M) with EBNA-1 IgG, VCA IgG, HHV-6 IgG, and HHV-6 IgM titers in samples collected prior to teriflunomide treatment (0M). A statistical significant association was found for VCA IgG (0M) with Nf-L measured at 0M (r = 0.221; p = 0.028) and at 6M (r = 0.240; p = 0.017). Correlations were assessed by using the Spearman’s rank correlation coefficient (r).

When we analyzed the correlation between the titers of each one of the viral antibodies prior to the initiation of teriflunomide treatment with the Nf-L levels for the basal sample and for the 6-month sample (**Figure 4**), we found a statistical significant correlation for VCA IgG in both cases: r = 0.221 (p = 0.028) for the basal sample and r = 0.240 (p = 0.017) for the 6-month sample.

### 3.3 Clinical and radiological response after 2 years of treatment with teriflunomide

A percentage of 68.9% (62/90) were clinical responders (no progression, no relapses), and 14.4% (13/90) had therapeutic failure (≥2 relapses and/or progression) after 2 years of teriflunomide treatment. A percentage of 45.1% (37/82) were NEDA-3 (no progression, no relapses, no new T2 lesions, no gadolinium-enhanced lesions) after 2 years of teriflunomide treatment. We did not find any statistical significant association between the variation of the titers of any of the viruses between the samples collected at 0M and 6M with the clinical response or the NEDA-3 condition after 2 years of treatment. Furthermore, the increase or decrease of the Nf-L levels was not also associated with the response to teriflunomide.

### 3.4 Demographic, clinical, radiological, and environmental data to identify early biomarkers of response to teriflunomide

When we analyzed the variables that could predict NEDA-3 conditions at treatment initiation, we found the following statistical associations in the univariate analysis (p < 0.05): starting age, disease duration, EBNA-1 titers, and number of gadolinium-enhanced lesions, all of them at the recruitment (before starting teriflunomide treatment). Therefore, those MS patients that were older, with long-lasting disease, higher EBNA-1 titers, and lower number of gadolinium-enhanced lesions were better responders. When we performed the multivariate analysis, only two variables retained the statistical signification: EBNA-1 titers (p = 0.001) and starting age of the treatment (p = 0.04).

Thus, among those MS patients with EBNA-1 titers ≥23.0 AU and ≥42.8 years at treatment initiation (median values of the NEDA-3 cohort), the 77.8% (14/18) were NEDA-3 vs. 37.5% (9/24) among those with both variables below the median value (p = 0.01; O.R. = 5.8). In our study, four MS patients were EBNA-1 negative (although VCA positive) and none of them were NEDA-3; furthermore, 3/4 EBNA-1 negative were therapeutic failure vs. 10/86 EBNA-1 positive (p = 0.02; O.R. = 6.5).

## 4 Discussion

In the last years, some authors have indicated that part of the benefits observed in MS patients treated with teriflunomide could be due to its antiviral properties. Gilli et al. evaluated the effect of teriflunomide in the Theiler model. The authors analyzed the effects of treatment on CNS viral load, intrathecal immune response, and disability progression. They observed that teriflunomide had both anti-inflammatory and antiviral properties, although they did not observe any impact on intrathecal antibody synthesis. Teriflunomide demonstrated a higher percentage of viral clearance compared with placebo, indicating that this treatment could have a prophylactic and therapeutic role against CNS viral infections (27). In our study, teriflunomide reduced the levels of IgG antibody titers against EBNA-1 and VCA of EBV and IgG and IgM against HHV-6 after 6 months of treatment in a real-life cohort of Spanish MS patients. These results are relevant since EBV has been proposed as the leading cause of MS (1). Our group recently published a paper in which we described that neither interferon-beta, glatiramer acetate, nor natalizumab had a significant effect on EBNA-1 and VCA IgG titers after 2 years of follow-up (28). In this previous study, we found that EBNA-1 IgG titers decreased in the 56.5% of patients treated with natalizumab, 39.1% of patients treated with interferon-beta, and 36.2% of patients treated with glatiramer acetate, after 6 months of treatment, which is significantly lower than the 73.3% found in the current cohort of MS patients treated with teriflunomide. The *in vivo* results of this study could be explained, at least in part, by the results of previous *in vitro* studies. In a paper published by Bilger et al. (8), teriflunomide inhibited cellular proliferation and promoted apoptosis, in EBV-transformed B cells *in vitro* at a clinically relevant dose. In addition, teriflunomide prevented the development of EBV-induced lymphomas in both a humanized mouse model and a xenograft model. Furthermore, teriflunomide inhibited lytic EBV infection *in vitro* both by preventing the initial steps of lytic viral reactivation and by blocking lytic viral DNA replication. Our results also support a previous *in vivo* study performed in a small cohort of MS patients. Zivadinov et al. (29) designed a longitudinal study with 30 MS who were starting treatment with teriflunomide. The authors found that MS patients experienced a significant decrease in IgG antibody titers against EBNA-1 (p = 0.003) and VCA (p = 0.05) after 12 months of treatment. MS patients who showed a greater decrease in IgG antibody titers against VCA and EBNA-1 from the baseline sample developed less loss of cortical volume (p < 0.001 and p = 0.02, respectively) and gray matter (p = 0.004 for both antibodies).

These antiviral properties could undoubtedly be of great interest in MS since, apart from the possible role of EBV in the etiopathogenesis of the disease, viral CNS infections are associated with other disease-modifying therapies (DMTs). It is the case of progressive multifocal leukoencephalopathy (PML) in which the causative agent is the JC virus (30–32). Given that teriflunomide can cross the blood–brain barrier (BBB) with 1–2% of serum concentrations (in the range of 2.5–4.1 μM) reaching the CNS (33), the usefulness of teriflunomide, at least in patients at risk of developing PML, could be hypothesized. This question has been addressed recently in a clinical trial (ClinicalTrials.gov Identifier: NCT01970410). In that study, authors assessed the safety and efficacy of rapid transition, from natalizumab to teriflunomide in MS patients. A total of 55 MS patients were enrolled, and 51% of them completed 24 months of teriflunomide. There were no cases of PML. The authors concluded that the washout-free transition of natalizumab to teriflunomide was an efficacious and safe strategy for patients at risk of developing PML (34).

In this study, we also evaluated the levels of Nf-L at basal visit and after 6 months of teriflunomide treatment. As we described above, we did not find any significant change in Nf-L levels. Since in the work of Bjornevik et al. (1) the authors assessed the possible temporal relation between EBV infection and Nf-L increase, we decided to analyze in our study the possible correlations between these two variables in the first 6 months with teriflunomide. In the previous study, it is described that NfL levels in EBV-negative MS patients at baseline were similar to those of controls before and around the time of EBV infection but increased after EBV infection. In our study, we found a correlation between VCA IgG titers at baseline and the Nf-L levels measured at the same time and 6 months later, but not with EBNA-1. This result is concordant with a previous study performed by Uher et al. (35); in this paper, authors found an association between VCA IgG levels and quartiles with sNfL levels, but not with EBNA-1. Thus, although more studies are needed to afford this question, EBV seems to be involved in those mechanisms that are associated with the release of Nf-L into the CSF and serum.

Finally, we analyzed the utility of different demographic, clinical, radiological, and environmental data to identify early biomarkers of response to teriflunomide. In the current MS scenario, with an increasing number of DMTs, it is crucial to identify the most appropriate treatment for each patient to ensure a good therapeutic response and to reduce costs. As we described previously, only EBNA-1 titers at baseline and the starting age of the treatment remain significant after the multivariate analysis. Those MS patients with both variables above the 50th percentile had a higher probability of reaching NEDA-3 conditions, since more than 75% of them were free of progression, relapses, new T2 lesions, and gadolinium-enhanced lesions, after 2 years of teriflunomide treatment. The results of the early biomarkers of response to teriflunomide highlight the possible antiviral role of this treatment since better results were obtained in MS patients with higher EBNA-1 IgG titers.

In conclusion, treatment with teriflunomide was associated with a reduction of the levels of IgG antibody titers against EBNA-1 and VCA of EBV and IgG and IgM against HHV-6 after 6 months of treatment in a real-life cohort of Spanish MS patients. A correlation between VCA IgG titers at baseline and the Nf-L levels measured at the same time and 6 months later was found. Furthermore, our results describe two variables that could be used as early predictors of NEDA-3 condition in MS patients that are going to initiate teriflunomide therapy.

## Data availability statement

The datasets presented in this study can be found in online repositories. The names of the repository/repositories and accession number(s) can be found in the article/**Supplementary Material**.

## Ethics statement

The studies involving humans were approved by Comité Ético Investigación Clínica del Hospital Clínico San Carlos. The studies were conducted in accordance with the local legislation and institutional requirements. The participants provided their written informed consent to participate in this study.

## Author contributions

MD-M prepared the samples, made the statistical analysis, discussed and interpreted findings, and revised the manuscript critically. MG-M prepared the samples and performed the ELISAs to detect antiviral antibodies. IG-S, LV, LC-F, NV, YA, BP, XM, MC, IC-P, MM-G, JG-D, and RA provided unique reagents, discussed and interpreted findings, and revised the manuscript critically. RA-L contributed to the design of the study, guided the progress of the study, and wrote the manuscript. All authors read and approved the final manuscript and have agreed both to be personally accountable for the author’s own contributions and to ensure that questions related to the accuracy or integrity of any part of the work, even ones in which the author was not personally involved, are appropriately investigated and resolved and the resolution documented in the literature.
