# Supplementary material for: Teriflunomide and Epstein–Barr virus in a Spanish multiple sclerosis cohort: in vivo antiviral activity and clinical response
Source: Front Immunol. 2023 Sep 29;14:1248182. doi: 10.3389/fimmu.2023.1248182 (PMC10570817; doi:10.3389/fimmu.2023.1248182)
Supplement: Supplementary file 1 [file Table_1.docx]

Supplementary Material

Teriflunomide and Epstein-Barr virus in a Spanish multiple sclerosis cohort: in vivo antiviral activity and clinical response

María Inmaculada Domínguez-Mozo^1^, Inés González-Suárez^2^, Luisa María Villar^3^, Lucienne Costa-Frossard^4^, Noelia Villarrubia^3^, Yolanda Aladro^5^, Belén Pilo^5^, Xavier Montalbán^6^, Manuel Comabella^6^, Ignacio Casanova-Peño^7^, María Luisa Martínez-Ginés^8^, Jose Manuel García-Domínguez^8^, María Ángel García-Martínez^1^, Rafael Arroyo^9^, Roberto Álvarez-Lafuente^1*^

*** Correspondence:** Dr. Roberto Álvarez Lafuente. Hospital Clínico San Carlos. Pabellón B. Laboratorio Investigación Esclerosis Múltiple. C/ Martín Lagos s/n. 28040 Madrid. España. Tel. +34-91-3303000. Ext. 482454. E-mail. ralvarezlafuente@yahoo.es.

# Supplementary Data

| **SAMPLES** | **GENDER** | **STARTING AGE OF THE DISEASE** | **AGE AT TREATMENT INITIATION** | **DURATION OF THE DISEASE (months)** | **T2 LESIONS AT TREATMENT INITIATION** | **GD+ LESIONS AT TREATMENT INITIATION** | **EDSS AT TREATMENT INITIATION** | **RELAPSES 2-YEARS BEFORE TREATMENT INITIATION** | **TREATMENT SUSPENDED BEFORE 24 MONTHS** | **NFL** | **IgG HHV-6 (AU/ml)** | **IgM HHV-6 (AU/ml)** | **IgG EBNA-1 EBV (AU/ml)** | **IgG VCA EBV (AU/ml)** |
| --- | --- | --- | --- | --- | --- | --- | --- | --- | --- | --- | --- | --- | --- | --- |
| 001B | 1 | 41,5 | 43,7 | 26,40 | 9 |  | 1,5 | 2 | 2 | 38,23 | 37,63 | 6,31 | 18,92 | 39,68 |
| 001S | 1 | 41,5 | 43,7 | 26,40 | 9 |  | 1,5 | 2 | 2 | 12,26 | 33,87 | 4,94 | 18,12 | 40,15 |
| 002B | 1 | 22,6 | 35,2 | 152,67 | 24 | 3 | 1,5 | 0 | 2 | 5,85 | 29,52 | 3,91 | 21,82 | 10,09 |
| 002S | 1 | 22,6 | 35,2 | 152,67 | 24 | 3 | 1,5 | 0 | 2 | 5,18 | 29,82 | 5,53 | 20,73 | 9,71 |
| 003B | 2 | 40,9 | 46,9 | 73,07 | 14 | 0 | 6 | 2 | 2 | 11,20 | 19,08 | 4,86 | 5,64 | 43,18 |
| 003S | 2 | 40,9 | 46,9 | 73,07 | 14 | 0 | 6 | 2 | 2 | 16,01 | 14,31 | 3,11 | 5,87 | 40,50 |
| 004B | 1 | 26,2 | 44,9 | 227,33 | 22 | 0 | 1,5 | 0 | 2 | 2,70 | 48,65 | 6,69 | 24,05 | 42,19 |
| 004S | 1 | 26,2 | 44,9 | 227,33 | 22 | 0 | 1,5 | 0 | 2 | 6,63 | 44,61 | 5,82 | 23,80 | 42,14 |
| 005B | 2 | 43,6 | 45,3 | 20,13 | 2 | 0 | 2 | 2 | 2 | 10,63 | 36,95 | 5,41 | 26,97 | 44,28 |
| 005S | 2 | 43,6 | 45,3 | 20,13 | 2 | 0 | 2 | 2 | 2 | 11,21 | 33,24 | 5,85 | 26,47 | 44,70 |
| 006B | 1 | 43,3 | 50,3 | 85,23 | 40 | 0 | 2 | 0 | 2 | 8,95 | 45,33 | 14,56 | 21,65 | 53,29 |
| 006S | 1 | 43,3 | 50,3 | 85,23 | 40 | 0 | 2 | 0 | 2 | 11,00 | 38,45 | 14,30 | 22,52 | 46,00 |
| 007B | 1 | 30,8 | 51,7 | 254,63 | 23 | 0 | 1,5 | 0 | 2 | 8,16 | 33,56 | 4,56 | 24,48 | 42,28 |
| 007S | 1 | 30,8 | 51,7 | 254,63 | 23 | 0 | 1,5 | 0 | 2 | 9,27 | 36,58 | 3,56 | 24,04 | 41,70 |
| 008B | 1 | 28,2 | 49,6 | 259,67 |  | 0 | 1,5 | 0 | 2 | 7,71 | 27,29 | 18,12 | 28,76 | 42,64 |
| 008S | 1 | 28,2 | 49,6 | 259,67 |  | 0 | 1,5 | 0 | 2 | 10,70 | 25,78 | 19,53 | 29,97 | 51,62 |
| 009B | 1 | 37,7 | 57,7 | 243,50 | 60 | 0 | 1,5 | 2 | 2 | 13,05 | 13,73 | 7,99 | 26,25 | 43,84 |
| 009S | 1 | 37,7 | 57,7 | 243,50 | 60 | 0 | 1,5 | 2 | 2 | 15,99 | 14,16 | 5,10 | 27,70 | 43,19 |
| 010B | 1 | 45,6 | 47,0 | 17,83 | 10 | 1 | 2 | 3 | 2 | 5,58 | 23,42 | 7,09 | 22,38 | 36,20 |
| 010S | 1 | 45,6 | 47,0 | 17,83 | 10 | 1 | 2 | 3 | 2 | 5,85 | 24,49 | 7,43 | 22,73 | 36,52 |
| 011B | 1 | 23,4 | 40,2 | 203,90 | 35 | 0 | 1,5 | 0 | 2 | 4,54 | 1,03 | 3,65 | 21,59 | 59,28 |
| 011S | 1 | 23,4 | 40,2 | 203,90 | 35 | 0 | 1,5 | 0 | 2 | 5,71 | 0,30 | 2,93 | 20,40 | 54,22 |
| 012B | 1 | 21,4 | 35,4 | 170,47 | 9 | 0 | 1 | 0 | 2 | 8,60 | 9,73 | 14,79 | 22,44 | 47,26 |
| 012S | 1 | 21,4 | 35,4 | 170,47 | 9 | 0 | 1 | 0 | 2 | 13,90 | 9,30 | 13,41 | 22,39 | 45,26 |
| 013B | 1 | 47,2 | 54,2 | 85,23 | 48 | 0 | 1,5 | 0 | 2 | 13,86 | 32,04 | 9,18 | 25,22 | 53,20 |
| 013S | 1 | 47,2 | 54,2 | 85,23 | 48 | 0 | 1,5 | 0 | 2 | 13,73 | 29,14 | 11,14 | 23,33 | 50,21 |
| 014B | 1 | 34,8 | 42,8 | 97,40 | 60 | 1 | 1,5 | 0 | 2 | 10,92 | 20,57 | 25,70 | 21,44 | 49,30 |
| 014S | 1 | 34,8 | 42,8 | 97,40 | 60 | 1 | 1,5 | 0 | 2 | 11,07 | 23,66 | 24,54 | 20,77 | 47,27 |
| 015B | 1 | 20,6 | 24,2 | 44,67 | 23 | 1 | 1,5 | 2 | 1 | 16,01 | 34,78 | 12,99 | 22,92 | 45,14 |
| 015S | 1 | 20,6 | 24,2 | 44,67 | 23 | 1 | 1,5 | 2 | 1 | 21,14 | 35,12 | 10,83 | 22,39 | 42,64 |
| 016B | 1 | 19,0 | 36,9 | 218,10 | 30 | 0 | 6,5 | 0 | 2 | 12,99 | 19,81 | 8,01 | 20,53 | 46,96 |
| 016S | 1 | 19,0 | 36,9 | 218,10 | 30 | 0 | 6,5 | 0 | 2 | 14,58 | 14,92 | 6,89 | 20,06 | 47,27 |
| 017B | 1 | 33,1 | 43,5 | 125,83 | 11 | 0 | 2,5 | 0 | 2 | 7,60 | 27,59 | 18,86 | 26,33 | 47,01 |
| 017S | 1 | 33,1 | 43,5 | 125,83 | 11 | 0 | 2,5 | 0 | 2 | 6,53 | 22,58 | 39,84 | 22,14 | 47,76 |
| 018B | 1 | 29,8 | 41,5 | 142,20 | 24 | 0 | 1,5 | 0 | 2 | 8,02 | 23,00 | 5,24 | 20,53 | 41,69 |
| 018S | 1 | 29,8 | 41,5 | 142,20 | 24 | 0 | 1,5 | 0 | 2 | 7,36 | 26,20 | 6,76 | 25,28 | 57,21 |
| 019B | 1 | 25,7 | 37,0 | 136,97 | 60 | 0 | 2 | 1 | 2 | 13,58 | 48,95 | 24,67 | 25,62 | 45,74 |
| 019S | 1 | 25,7 | 37,0 | 136,97 | 60 | 0 | 2 | 1 | 2 | 24,64 | 48,51 | 27,05 | 24,62 | 45,32 |
| 020B | 2 | 30,8 | 32,6 | 21,27 | 17 | 0 | 1 | 1 | 2 | 6,67 | 31,06 | 1,37 | 14,51 | 41,69 |
| 020S | 2 | 30,8 | 32,6 | 21,27 | 17 | 0 | 1 | 1 | 2 | 6,93 | 30,07 | 1,16 | 13,33 | 40,42 |
| 021B | 2 | 37,7 | 43,4 | 69,97 | 50 | 0 | 2 | 2 | 1 | 22,82 | 37,14 | 31,61 | 22,91 | 72,90 |
| 021S | 2 | 37,7 | 43,4 | 69,97 | 50 | 0 | 2 | 2 | 1 | 21,23 | 33,19 | 26,51 | 22,22 | 69,14 |
| 022B | 1 | 27,2 | 57,7 | 370,30 | 120 | 0 | 3,5 | 0 | 2 | 16,14 | 16,43 | 9,76 | 23,21 | 57,57 |
| 022S | 1 | 27,2 | 57,7 | 370,30 | 120 | 0 | 3,5 | 0 | 2 | 44,58 | 10,60 | 8,21 | 22,31 | 62,38 |
| 023B | 1 | 36,8 | 44,0 | 88,27 | 60 | 0 | 2 | 0 | 2 | 11,01 | 30,98 | 10,63 | 17,83 | 47,65 |
| 023S | 1 | 36,8 | 44,0 | 88,27 | 60 | 0 | 2 | 0 | 2 | 12,10 | 30,26 | 8,37 | 16,99 | 48,58 |
| 024B | 1 | 33,8 | 45,7 | 144,47 | 54 | 0 | 1,5 | 0 | 1 | 38,99 | 50,49 | 12,45 | 5,76 | 53,66 |
| 024S | 1 | 33,8 | 45,7 | 144,47 | 54 | 0 | 1,5 | 0 | 1 | 19,18 | 42,02 | 7,79 | 6,99 | 67,65 |
| 025B | 2 | 41,4 | 47,5 | 74,07 | 60 | 0 | 1,5 | 0 | 2 | 12,03 | 15,71 | 3,71 | 23,43 | 42,08 |
| 025S | 2 | 41,4 | 47,5 | 74,07 | 60 | 0 | 1,5 | 0 | 2 | 9,50 | 18,12 | 5,24 | 23,16 | 45,35 |
| 026B | 1 | 27,0 | 40,8 | 167,40 | 23 | 0 | 1,5 | 1 | 2 | 38,62 | 42,06 | 6,50 | 16,68 | 46,39 |
| 026S | 1 | 27,0 | 40,8 | 167,40 | 23 | 0 | 1,5 | 1 | 2 | 9,41 | 44,61 | 4,06 | 16,75 | 46,75 |
| 027B | 2 | 29,9 | 35,8 | 71,07 | 11 | 0 | 5 | 0 | 2 | 6,00 | 24,28 | 4,36 | 25,06 | 36,79 |
| 027S | 2 | 29,9 | 35,8 | 71,07 | 11 | 0 | 5 | 0 | 2 | 8,30 | 17,30 | 2,72 | 23,95 | 33,44 |
| 028B | 1 | 19,2 | 20,0 | 9,30 | 20 | 0 | 1 | 1 | 2 | 12,46 | 33,28 | 5,24 | 28,01 | 44,42 |
| 028S | 1 | 19,2 | 20,0 | 9,30 | 20 | 0 | 1 | 1 | 2 | 7,32 | 31,47 | 3,24 | 25,83 | 43,62 |
| 029B | 2 | 27,8 | 59,7 | 387,57 | 35 | 0 | 3,5 | 1 | 2 | 17,75 | 44,90 | 13,68 | 26,53 | 51,11 |
| 029S | 2 | 27,8 | 59,7 | 387,57 | 35 | 0 | 3,5 | 1 | 2 | 14,06 | 35,75 | 10,45 | 26,88 | 49,34 |
| 030B | 2 | 58,9 | 59,2 | 3,03 | 40 | 1 | 1 | 1 | 2 | 14,57 | 39,31 | 12,55 | 21,85 | 48,85 |
| 030S | 2 | 58,9 | 59,2 | 3,03 | 40 | 1 | 1 | 1 | 2 | 16,84 | 40,85 | 12,00 | 21,56 | 47,31 |
| 031B | 2 | 46,6 | 47,8 | 14,17 | 3 | 0 | 1,5 | 3 | 2 | 8,10 | 27,65 | 2,66 | 25,82 | 43,01 |
| 031S | 2 | 46,6 | 47,8 | 14,17 | 3 | 0 | 1,5 | 3 | 2 | 17,70 | 27,65 | 1,94 | 25,47 | 42,40 |
| 032B | 2 | 32,2 | 41,0 | 107,60 | 6 | 1 | 6 | 0 | 1 | 4,79 | 37,58 | 8,55 | 19,78 | 24,78 |
| 032S | 2 | 32,2 | 41,0 | 107,60 | 6 | 1 | 6 | 0 | 1 | 6,17 | 41,10 | 8,33 | 18,03 | 27,86 |
| 033B | 1 | 24,3 | 34,5 | 124,80 | 28 | 0 | 2,5 | 1 | 2 | 7,75 | 32,69 | 7,25 | 23,37 | 54,12 |
| 033S | 1 | 24,3 | 34,5 | 124,80 | 28 | 0 | 2,5 | 1 | 2 | 5,08 | 24,48 | 5,70 | 22,62 | 51,35 |
| 034B | 1 | 30,3 | 41,5 | 135,93 | 60 | 0 | 1 | 1 | 2 | 8,33 | 24,23 | 4,84 | 27,19 | 46,75 |
| 034S | 1 | 30,3 | 41,5 | 135,93 | 60 | 0 | 1 | 1 | 2 | 14,74 | 22,98 | 6,01 | 27,59 | 45,74 |
| 035B | 1 | 31,3 | 50,2 | 230,30 | 19 | 0 | 1,5 | 0 | 2 | 17,23 | 35,26 | 2,48 | 19,34 | 45,69 |
| 035S | 1 | 31,3 | 50,2 | 230,30 | 19 | 0 | 1,5 | 0 | 2 | 28,34 | 31,64 | 1,70 | 18,86 | 42,86 |
| 036B | 1 | 30,2 | 33,1 | 34,57 | 39 | 4 | 0 | 2 | 2 | 16,70 | 9,17 | 9,00 | 20,55 | 38,83 |
| 036S | 1 | 30,2 | 33,1 | 34,57 | 39 | 4 | 0 | 2 | 2 | 12,75 | 12,91 | 6,66 | 20,81 | 38,90 |
| 037B | 2 | 27,2 | 42,4 | 184,93 | 19 | 1 | 2,5 | 1 | 2 | 10,69 | 31,35 | 5,28 | 21,95 | 69,18 |
| 037S | 2 | 27,2 | 42,4 | 184,93 | 19 | 1 | 2,5 | 1 | 2 | 8,32 | 32,93 | 2,98 | 21,81 | 53,86 |
| 038B | 2 | 22,7 | 44,4 | 264,83 | 15 | 0 | 1,5 | 0 | 1 | 9,82 | 24,43 | 1,96 | 27,15 | 47,12 |
| 038S | 2 | 22,7 | 44,4 | 264,83 | 15 | 0 | 1,5 | 0 | 1 | 13,89 | 17,68 | 2,00 | 25,92 | 47,17 |
| 039B | 1 | 33,6 | 47,7 | 171,50 | 16 | 0 | 2 | 1 | 2 | 16,97 | 37,77 | 1,90 | 21,48 | 48,44 |
| 039S | 1 | 33,6 | 47,7 | 171,50 | 16 | 0 | 2 | 1 | 2 | 13,71 | 35,02 | 2,10 | 21,58 | 48,64 |
| 040B | 1 | 39,5 | 43,9 | 52,77 | 24 | 0 | 1,5 | 1 | 2 | 9,71 | 19,37 | 4,90 | 19,81 | 49,73 |
| 040S | 1 | 39,5 | 43,9 | 52,77 | 24 | 0 | 1,5 | 1 | 2 | 8,18 | 13,59 | 3,71 | 19,25 | 49,44 |
| 041B | 1 | 38,8 | 53,8 | 181,57 | 30 | 0 | 5 | 1 | 2 | 7,19 | 25,15 | 3,91 | 16,18 | 43,47 |
| 041S | 1 | 38,8 | 53,8 | 181,57 | 30 | 0 | 5 | 1 | 2 | 10,94 | 22,50 | 2,14 | 15,32 | 43,05 |
| 042B | 1 | 20,5 | 57,2 | 446,37 | 26 | 0 | 1,5 | 0 | 2 | 10,31 | 11,08 | 5,06 | 25,65 | 48,23 |
| 042S | 1 | 20,5 | 57,2 | 446,37 | 26 | 0 | 1,5 | 0 | 2 | 12,85 | 13,40 | 6,04 | 25,56 | 69,17 |
| 043B | 1 | 41,6 | 42,8 | 14,17 | 3 | 0 | 0 | 2 | 2 | 7,85 | 18,55 | 4,24 | 11,48 | 14,87 |
| 043S | 1 | 41,6 | 42,8 | 14,17 | 3 | 0 | 0 | 2 | 2 | 6,76 | 16,26 | 3,98 | 12,71 | 14,57 |
| 044B | 1 | 28,9 | 41,0 | 147,10 | 38 | 1 | 1,5 | 0 | 2 | 19,50 | 35,78 | 5,24 | 24,20 | 42,95 |
| 044S | 1 | 28,9 | 41,0 | 147,10 | 38 | 1 | 1,5 | 0 | 2 | 28,64 | 39,45 | 2,84 | 23,94 | 40,72 |
| 045B | 1 | 45,0 | 46,6 | 20,27 | 25 | 0 | 1,5 | 1 | 2 | 5,23 | 38,20 | 6,19 | 23,09 | 44,48 |
| 045S | 1 | 45,0 | 46,6 | 20,27 | 25 | 0 | 1,5 | 1 | 2 | 9,01 | 42,54 | 5,71 | 21,79 | 43,14 |
| 046B | 1 | 42,8 | 43,4 | 7,30 | 9 | 0 | 2 | 1 | 2 | 14,40 | 30,30 | 4,52 | 22,23 | 41,97 |
| 046S | 1 | 42,8 | 43,4 | 7,30 | 9 | 0 | 2 | 1 | 2 | 7,66 | 30,79 | 3,85 | 22,22 | 42,27 |
| 047B | 1 | 33,1 | 49,0 | 193,80 | 25 | 2 | 1,5 | 0 | 2 | 6,31 | 5,25 | 2,52 | 19,00 | 42,88 |
| 047S | 1 | 33,1 | 49,0 | 193,80 | 25 | 2 | 1,5 | 0 | 2 | 6,01 | 5,59 | 1,63 | 19,80 | 43,80 |
| 048B | 1 | 36,8 | 46,2 | 114,70 | 60 | 1 | 2,5 | 0 | 2 | 13,88 | 22,11 | 5,10 | 22,34 | 46,94 |
| 048S | 1 | 36,8 | 46,2 | 114,70 | 60 | 1 | 2,5 | 0 | 2 | 13,01 | 25,00 | 4,54 | 21,64 | 46,21 |
| 049B | 1 | 27,3 | 47,1 | 241,47 | 37 | 0 | 1,5 | 0 | 2 | 18,68 | 39,60 | 16,80 | 11,97 | 45,39 |
| 049S | 1 | 27,3 | 47,1 | 241,47 | 37 | 0 | 1,5 | 0 | 2 | 13,26 | 37,53 | 11,01 | 10,66 | 44,92 |
| 050B | 1 | 38,5 | 43,3 | 58,83 | 6 | 0 | 1 | 0 | 2 | 5,87 | 39,36 | 51,26 | 27,66 | 43,29 |
| 050S | 1 | 38,5 | 43,3 | 58,83 | 6 | 0 | 1 | 0 | 2 | 10,77 | 27,12 | 11,38 | 25,34 | 38,97 |
| 051B | 2 | 37,8 | 38,3 | 6,13 | 25 | 1 | 1,5 | 1 | 2 | 27,29 | 19,85 | 9,02 | 25,41 | 28,76 |
| 051S | 2 | 37,8 | 38,3 | 6,13 | 25 | 1 | 1,5 | 1 | 2 | 9,17 | 19,46 | 4,86 | 25,04 | 28,12 |
| 052B | 1 | 19,3 | 25,0 | 69,03 | 11 | 2 | 1 | 0 | 2 | 5,23 | 34,49 | 6,92 | 15,85 | 33,51 |
| 052S | 1 | 19,3 | 25,0 | 69,03 | 11 | 2 | 1 | 0 | 2 | 7,43 | 34,54 | 5,71 | 14,30 | 33,10 |
| 053B | 2 | 22,8 | 30,1 | 88,23 | 50 | 0 | 3,5 | 1 | 2 | 7,86 | 8,29 | 0,61 | 22,24 | 44,12 |
| 053S | 2 | 22,8 | 30,1 | 88,23 | 50 | 0 | 3,5 | 1 | 2 | 11,45 | 5,73 | 0,52 | 21,89 | 43,16 |
| 054B | 2 | 29,5 | 36,3 | 83,20 | 11 | 0 | 1,5 | 2 | 1 | 7,65 | 36,08 | 7,99 | 23,00 | 41,12 |
| 054S | 2 | 29,5 | 36,3 | 83,20 | 11 | 0 | 1,5 | 2 | 1 | 7,13 | 33,16 | 8,01 | 23,88 | 45,67 |
| 055B | 1 | 26,6 | 44,2 | 214,10 | 18 | 0 | 3,5 | 2 | 1 | 17,74 | 29,73 | 4,06 | 20,14 | 45,47 |
| 055S | 1 | 26,6 | 44,2 | 214,10 | 18 | 0 | 3,5 | 2 | 1 | 17,50 | 30,59 | 2,89 | 20,22 | 45,31 |
| 056B | 1 | 16,3 | 22,3 | 73,07 | 5 | 0 | 1,5 | 1 | 2 | 6,12 | 40,04 | 6,58 | 14,96 | 42,83 |
| 056S | 1 | 16,3 | 22,3 | 73,07 | 5 | 0 | 1,5 | 1 | 2 | 6,19 | 42,01 | 3,87 | 12,79 | 42,33 |
| 057B | 1 | 28,5 | 39,2 | 129,80 | 17 | 0 | 1,5 | 0 | 2 | 5,86 | 28,42 | 2,62 | 20,80 | 37,15 |
| 057S | 1 | 28,5 | 39,2 | 129,80 | 17 | 0 | 1,5 | 0 | 2 | 5,18 | 28,38 | 2,12 | 20,71 | 37,19 |
| 058B | 1 | 37,1 | 38,5 | 17,27 | 60 | 0 | 2 | 1 | 2 | 14,54 | 29,92 | 9,04 | 13,79 | 41,46 |
| 058S | 1 | 37,1 | 38,5 | 17,27 | 60 | 0 | 2 | 1 | 2 | 6,36 | 30,01 | 9,08 | 12,68 | 40,16 |
| 059B | 2 | 56,0 | 57,4 | 17,23 | 9 | 1 | 6 | 3 | 2 | 7,30 | 0,58 | 5,35 | 16,81 | 42,38 |
| 059S | 2 | 56,0 | 57,4 | 17,23 | 9 | 1 | 6 | 3 | 2 | 9,80 | 0,67 | 6,54 | 15,88 | 41,11 |
| 060B | 1 | 40,4 | 40,9 | 6,03 | 27 | 5 | 2 | 2 | 1 | 11,76 | 19,32 | 5,08 | 18,49 | 42,84 |
| 060S | 1 | 40,4 | 40,9 | 6,03 | 27 | 5 | 2 | 2 | 1 | 13,14 | 24,36 | 3,79 | 18,22 | 42,13 |
| 061B | 1 | 29,4 | 30,7 | 15,23 | 5 | 3 | 1 | 2 | 2 | 5,22 | 27,16 | 7,77 | 21,71 | 41,18 |
| 061S | 1 | 29,4 | 30,7 | 15,23 | 5 | 3 | 1 | 2 | 2 | 5,29 | 28,79 | 8,77 | 24,45 | 48,12 |
| 062B | 1 | 30,1 | 39,7 | 116,63 | 33 | 0 | 1 | 0 | 2 | 6,23 | 43,22 | 7,87 | 21,75 | 34,62 |
| 062S | 1 | 30,1 | 39,7 | 116,63 | 33 | 0 | 1 | 0 | 2 | 6,06 | 49,02 | 6,17 | 21,91 | 33,01 |
| 063B | 1 | 26,1 | 32,3 | 75,40 | 11 | 2 | 1,5 | 1 | 2 | 10,01 | 56,68 | 4,68 | 13,46 | 43,00 |
| 063S | 1 | 26,1 | 32,3 | 75,40 | 11 | 2 | 1,5 | 1 | 2 | 9,47 | 50,58 | 4,36 | 12,90 | 56,25 |
| 064B | 1 | 24 | 33,9 | 139 | 60 | 0 | 2 | 0 | 1 | 9,48 | 25,61 | 12,68 | 31,93 | 32,18 |
| 064S | 1 | 24 | 33,9 | 139 | 60 | 0 | 2 | 0 | 1 | 14,38 | 26,73 | 9,49 | 31,42 | 29,61 |
| 065B | 1 | 24 | 50,6 | 298 | 60 | 0 | 3,5 | 0 | 2 | 9,75 | 9,52 | 1,00 | 3,79 | 52,45 |
| 065S | 1 | 24 | 50,6 | 298 | 60 | 0 | 3,5 | 0 | 2 | 18,37 | 6,95 | 0,79 | 3,71 | 52,67 |
| 066B | 2 |  | 40,2 | 186 | 45 | 0 | 1 | 1 | 2 | 10,68 | 39,58 | 6,72 | 25,42 | 37,07 |
| 066S | 2 |  | 40,2 | 186 | 45 | 0 | 1 | 1 | 2 | 9,80 | 36,53 | 3,51 | 25,03 | 37,27 |
| 067B | 2 | 14 | 44,4 | 10 | 4 | 0 | 4 | 1 | 2 | 7,28 | 12,69 | 4,55 | 23,82 | 25,65 |
| 067S | 2 | 14 | 44,4 | 10 | 4 | 0 | 4 | 1 | 2 | 9,67 | 9,49 | 3,31 | 23,75 | 24,76 |
| 068B | 1 |  | 36,4 | 64 | 60 | 0 | 1,5 | 2 | 2 | 10,12 | 45,02 | 10,40 | 18,72 | 50,44 |
| 068S | 1 |  | 36,4 | 64 | 60 | 0 | 1,5 | 2 | 2 | 7,68 | 42,90 | 9,35 | 20,38 | 53,12 |
| 069B | 1 | 18 | 42,0 | 230 | 60 |  | 2 | 0 | 2 | 5,19 | 29,20 | 2,86 | 25,15 | 47,61 |
| 069S | 1 | 18 | 42,0 | 230 | 60 |  | 2 | 0 | 2 | 6,20 | 32,40 | 4,18 | 23,68 | 47,82 |
| 070B | 2 | 27 | 40,1 | 168 | 45 | 0 | 4 | 1 | 2 |  | 35,59 | 5,52 | 21,40 | 46,94 |
| 070S | 2 | 27 | 40,1 | 168 | 45 | 0 | 4 | 1 | 2 |  | 38,78 | 2,36 | 20,70 | 44,38 |
| 071B | 1 | 19 | 50,4 | 192 | 60 | 0 | 2 | 0 | 2 | 7,85 | 36,53 | 5,81 | 12,61 | 50,00 |
| 071S | 1 | 19 | 50,4 | 192 | 60 | 0 | 2 | 0 | 2 | 6,36 | 35,11 | 4,71 | 10,05 | 47,60 |
| 072B | 1 |  | 40,5 | 92 | 9 | 0 | 2 | 1 | 2 | 10,18 | 35,10 | 5,76 | 28,37 | 56,88 |
| 072S | 1 |  | 40,5 | 92 | 9 | 0 | 2 | 1 | 2 | 10,32 | 35,28 | 2,53 | 28,86 | 54,38 |
| 073B | 1 |  | 49,5 | 51 | 60 | 2 | 1 | 0 | 1 | 17,66 | 25,04 | 4,59 | 27,71 | 42,40 |
| 073S | 1 |  | 49,5 | 51 | 60 | 2 | 1 | 0 | 1 | 11,47 | 23,22 | 3,25 | 25,84 | 39,03 |
| 074B | 1 | 27 | 20,7 | 19 | 45 | 0 | 1,5 | 2 | 2 | 6,55 | 42,95 | 18,10 | 23,63 | 44,74 |
| 074S | 1 | 27 | 20,7 | 19 | 45 | 0 | 1,5 | 2 | 2 | 6,06 | 39,08 | 14,54 | 23,93 | 42,32 |
| 075B | 2 | 45 | 42,8 | 26 | 45 | 0 | 1 | 0 | 2 | 8,61 | 18,97 | 2,10 | 26,32 | 52,32 |
| 075S | 2 | 45 | 42,8 | 26 | 45 | 0 | 1 | 0 | 2 | 7,97 | 20,52 | 3,55 | 25,22 | 50,91 |
| 076B | 1 | 22 | 40,4 | 55 | 45 | 4 | 2 | 1 | 1 | 25,04 | 12,70 | 12,04 | 10,65 | 52,73 |
| 076S | 1 | 22 | 40,4 | 55 | 45 | 4 | 2 | 1 | 1 | 16,60 | 5,87 | 7,60 | 7,18 | 49,67 |
| 077B | 1 | 45 | 42,9 | 90 | 100 | 0 | 2 | 0 | 2 | 8,38 | 33,11 | 13,49 | 23,99 | 55,82 |
| 077S | 1 | 45 | 42,9 | 90 | 100 | 0 | 2 | 0 | 2 | 9,86 | 30,43 | 12,30 | 23,03 | 55,30 |
| 078B | 1 | 26 | 41,3 | 247 | 50 | 0 | 1,5 | 1 | 2 | 8,50 | 31,62 | 8,45 | 28,40 | 50,13 |
| 078S | 1 | 26 | 41,3 | 247 | 50 | 0 | 1,5 | 1 | 2 | 7,78 | 36,87 | 5,92 | 29,19 | 49,20 |
| 079B | 1 |  | 48,9 | 107 | 60 | 0 | 2 | 1 | 2 | 6,61 | 39,88 | 2,28 | 24,32 | 48,16 |
| 079S | 1 |  | 48,9 | 107 | 60 | 0 | 2 | 1 | 2 | 10,25 | 17,74 | 10,95 | 22,08 | 53,93 |
| 080B | 1 | 23 | 55,4 | 237 | 120 | 1 | 6 | 1 | 1 | 12,55 | 41,27 | 6,45 | 24,68 | 57,55 |
| 080S | 1 | 23 | 55,4 | 237 | 120 | 1 | 6 | 1 | 1 | 11,22 | 41,81 | 3,05 | 24,86 | 56,58 |
| 081B | 2 | 21 | 37,4 | 30 | 60 | 2 | 1,5 | 0 | 1 | 6,92 | 30,97 | 6,76 | 19,48 | 13,17 |
| 081S | 2 | 21 | 37,4 | 30 | 60 | 2 | 1,5 | 0 | 1 | 22,40 | 31,60 | 6,87 | 18,32 | 12,20 |
| 082B | 1 | 46 | 58 | 143 | 47 |  | 2 | 0 | 2 | 8,17 | 19,00 | 17,14 | 9,57 | 68,64 |
| 082S | 1 | 46 | 58 | 143 | 47 |  | 2 | 0 | 2 | 12,15 | 20,86 | 10,47 | 6,63 | 52,67 |
| 083B | 1 | 30 | 38 | 101 | 50 | 0 | 1 | 0 | 2 | 12,03 | 49,48 | 11,61 | 22,47 |  |
| 083S | 1 | 30 | 38 | 101 | 50 | 0 | 1 | 0 | 2 | 12,02 | 49,53 | 11,10 | 22,18 | 34,19 |
| 084B | 1 | 23 | 53 | 367 | 64 | 0 | 0 | 0 | 2 | 8,55 | 28,03 | 10,40 | 23,85 | 68,65 |
| 084S | 1 | 23 | 53 | 367 | 64 | 0 | 0 | 0 | 2 | 12,96 | 27,68 | 9,31 | 23,29 | 68,60 |
| 085B | 2 | 38 | 41 | 36 | 45 | 0 | 0 | 0 | 2 | 5,99 | 32,28 | 24,65 | 22,80 | 0,05 |
| 085S | 2 | 38 | 41 | 36 | 45 | 0 | 0 | 0 | 2 | 12,28 | 28,32 | 20,80 | 22,46 | 2,93 |
| 086B | 1 | 34 | 36 | 19 | 45 | 0 | 2 | 2 | 2 | 5,70 | 15,09 | 3,33 | 0,30 | 49,80 |
| 086S | 1 | 34 | 36 | 19 | 45 | 0 | 2 | 2 | 2 | 8,96 | 11,95 | 2,62 | 0,23 | 48,49 |
| 087B | 1 | 39 | 41 | 22 | 24 | 0 | 1,5 | 1 | 2 | 3,89 | 35,61 | 19,08 | 26,56 | 50,79 |
| 087S | 1 | 39 | 41 | 22 | 24 | 0 | 1,5 | 1 | 2 | 5,04 | 34,85 | 13,13 | 25,94 | 51,84 |
| 088B | 1 | 32 | 52 | 246 |  |  | 2 | 1 | 2 | 9,94 | 19,58 | 7,20 | 23,22 | 47,25 |
| 088S | 1 | 32 | 52 | 246 |  |  | 2 | 1 | 2 | 8,27 | 19,52 | 9,06 | 23,15 | 46,68 |
| 089B | 1 | 41 | 57 | 184 |  |  | 3,5 | 0 | 2 | 16,04 | 20,28 | 52,64 | 14,15 | 46,02 |
| 089S | 1 | 41 | 57 | 184 |  |  | 3,5 | 0 | 2 | 17,76 | 13,35 | 31,92 | 13,44 | 44,80 |
| 090B | 1 | 37 | 51 | 170 |  |  | 3 | 0 | 2 | 7,12 | 36,71 | 5,24 | 20,80 | 48,04 |
| 090S | 1 | 37 | 51 | 170 |  |  | 3 | 0 | 2 | 10,30 | 33,04 | 5,01 | 20,22 | 47,12 |
| 091B | 1 | 33 | 33 | 5 |  | 1 | 0 | 1 | 2 | 6,75 | 33,63 | 9,55 | 23,29 | 36,36 |
| 091S | 1 | 33 | 33 | 5 |  | 1 | 0 | 1 | 2 | 7,64 | 29,66 | 8,01 | 22,72 | 35,92 |
| 092B | 2 | 45 | 45 | 5 |  | 3 | 0 | 1 | 2 | 12,45 | 41,78 | 5,95 | 22,68 | 47,40 |
| 092S | 2 | 45 | 45 | 5 |  | 3 | 0 | 1 | 2 | 24,02 | 44,00 | 5,91 | 23,18 | 44,74 |
| 093B | 1 | 43 | 54 | 133 |  |  | 2 | 0 | 2 | 8,50 | 18,82 | 14,68 | 20,92 | 48,22 |
| 093S | 1 | 43 | 54 | 133 |  |  | 2 | 0 | 2 | 13,45 | 14,22 | 11,83 | 20,09 | 46,45 |
| 094B | 2 | 38 | 38 | 7 | 10 | 0 | 0 | 1 | 2 | 8,25 | 31,29 | 7,36 | 22,33 | 48,66 |
| 094S | 2 | 38 | 38 | 7 | 10 | 0 | 0 | 1 | 2 | 8,48 | 38,93 | 7,94 | 21,30 | 44,95 |
| 095B | 1 | 51 | 55 | 38 |  | 2 | 2 | 3 | 2 | 6,06 | 40,91 | 16,31 | 20,75 | 45,91 |
| 095S | 1 | 51 | 55 | 38 |  | 2 | 2 | 3 | 2 | 10,76 | 38,93 | 10,78 | 20,33 | 44,32 |
| 096B | 1 | 29 | 51 | 264 | 47 |  | 2 | 0 | 2 | 10,05 | 34,85 | 8,83 | 21,84 | 51,61 |
| 096S | 1 | 29 | 51 | 264 | 47 |  | 2 | 0 | 2 | 12,97 | 32,93 | 17,24 | 23,29 | 52,54 |
| 097B | 2 | 23 | 51 | 346 | 45 |  | 2 | 0 | 2 | 7,95 | 0,57 | 1,50 | 20,58 | 55,55 |
| 097S | 2 | 23 | 51 | 346 | 45 |  | 2 | 0 | 2 | 10,28 | 0,53 | 1,71 | 21,35 | 55,09 |
| 098B | 1 | 38 | 41 | 12 | 26 | 0 | 0 | 0 | 2 | 8,29 | 22,05 | 5,36 | 9,92 | 41,74 |
| 098S | 1 | 38 | 41 | 12 | 26 | 0 | 0 | 0 | 2 | 8,28 | 18,54 | 2,92 | 9,98 | 38,41 |
| 099B | 1 | 27 | 27 | 10 | 6 |  | 2 |  | 2 | 10,04 | 33,31 | 24,53 | 24,07 | 39,76 |
| 099S | 1 | 27 | 27 | 10 | 6 |  | 2 |  | 2 | 8,01 | 35,52 | 8,05 | 22,36 | 37,53 |
| 100B | 2 | 34 | 44 | 120 | 30 |  | 2 | 0 | 2 | 4,97 | 28,16 | 1,18 | 21,41 | 52,57 |
| 100S | 2 | 34 | 44 | 120 | 30 |  | 2 | 0 | 2 | 6,19 | 27,91 | 1,18 | 20,30 | 51,03 |
| 101B | 1 | 29 | 49 | 237 |  |  | 2 | 0 | 2 |  | 33,68 | 2,87 | 25,09 | 51,96 |
| 101S | 1 | 29 | 49 | 237 |  |  | 2 | 0 | 2 |  | 37,62 | 2,90 | 24,73 | 51,89 |
